# Living Donor Liver Transplantation for Hepatic Venoocclusive Disease/Sinusoidal Obstruction Syndrome Originating from Hematopoietic Stem Cell Transplantation

**DOI:** 10.1155/2022/8361769

**Published:** 2022-05-21

**Authors:** Kentaro Ichimura, Norio Kawamura, Ryoichi Goto, Masaaki Watanabe, Yoshikazu Ganchiku, Tsuyoshi Shimamura, Akinobu Taketomi

**Affiliations:** ^1^Department of Gastroenterological Surgery I, Hokkaido University Hospital, Japan; ^2^Department of Transplant Surgery, Hokkaido University Graduate School of Medicine, Japan; ^3^Division of Organ Transplantation, Hokkaido University Hospital, Japan

## Abstract

**Background:**

Venoocclusive disease (VOD), also known as sinusoidal obstruction syndrome (SOS), is a life-threatening hematopoietic stem cell transplantation (HSCT) complication. Cases of mild and moderate VOD/SOS are self-limiting; however, the mortality for severe VOD/SOS has reached 80%. Recently, defibrotide became available and has been used for VOD/SOS; however, the outcome for patients with severe VOD/SOS is not satisfactory, and liver transplantation is attempted in these severe cases.

**Method:**

We describe a case of living donor liver transplantation (LDLT) for acute liver failure secondary to VOD/SOS that originates from HSCT.

**Result:**

Liver regeneration after LDLT was impaired, and several infections were developed before liver regeneration completion. Our patient suffered sepsis and finally died of multiorgan failure.

**Conclusion:**

Severe VOD/SOS originating from HSCT is associated with a very poor prognosis. The liver transplantation outcome for VOD/SOS has not been satisfied, but it may provide long-term survival if successful. We considered liver transplantation as a therapeutic option, especially in cases where sufficient graft volume is secured, considering impaired liver regeneration under bone marrow suppression after HSCT.

## 1. Introduction

Venoocclusive disease (VOD), also known as sinusoidal obstruction syndrome (SOS), is a life-threatening hematopoietic stem cell transplantation (HSCT) complication. A myeloablative preparative regimen, using busulfan (BU), cyclophosphamide (CY), and total body irradiation (TBI), increases the risk of VOD/SOS [1]. Cases of mild and moderate VOD/SOS are self-limiting; however, the mortality for severe VOD/SOS has reached 80% [1, 2]. Recently, defibrotide became available and has been used for VOD/SOS. However, satisfactory results have not yet been achieved with defibrotide in cases with severe VOD/SOS, such as those with acute liver failure, and liver transplantation is attempted in these severe cases. Here, we describe a case of living donor liver transplantation (LDLT) for acute liver failure secondary to VOD/SOS originating from HSCT.

## 2. Case Report

A 7-year-old female patient has been initially diagnosed with B-cell precursor acute lymphoblastic leukemia (B-ALL) with hyperdiploidy, which was known to have a favorable prognosis. She underwent combination chemotherapy and achieved initial complete remission. Relapse occurred 6 years following the completion of 2-year chemotherapy, after which combination therapy was repeated, and a second complete remission was achieved. Allogeneic HSCT from a human leukocyte antigen- (HLA-) matched sibling donor was planned when she was 16 years old. Myeloablative preparative chemoradiotherapy, including etoposide at 1800 mg/sqm, CY at 120 mg/kg, and TBI at 12 Gy in six fractions, was performed before HSCT. The patient received an intravenous infusion of bone marrow cells (a total of 3.27 × 10^8^/kg, including 2.3 × 10^6^/kg as CD34+ cells). Short-term methotrexate (15 mg on day +1 and 10 mg on day +3, +6, and +11) and cyclosporine A (3.0 mg/kg) daily from day -1 were used for graft-versus-host disease (GVHD).

On day +17 after HSCT, full-donor chimerism was detected using short tandem repeat analysis. However, she suffered weight gain, ascites, and hepatomegaly and had elevated liver enzymes on day +20 after HSCT. According to the European Society for Blood and Marrow Transplantation (EBMT) criteria, the diagnosis was VOD/SOS, moderate severity. Recombinant human soluble thrombomodulin (rhTM) therapy could not improve liver function.

On day +44 after HSCT, grade II hepatic encephalopathy and coagulopathy with a prothrombin time-international normalized ratio level of 3.0 developed, and the patient was diagnosed with acute liver failure secondary to VOD/SOS. VOD/SOS severity progressed to moderate to very severe. Defibrotide was unapproved in Japan at that time (2019/06-approved); thus, there were no other treatment options other than liver transplantation. Based on the achievement of complete chimera and an estimated 2-year recurrence rate of 0% [3], the decision was made to perform LDLT. Preoperative bone marrow aspiration revealed engraftment achievement, but lab tests showed pancytopenia despite daily GCSF administration (hemoglobin [Hb] of 7.7 g/dL, leukocytes of 3,700/L, and platelets of 49,000/L). LDLT was performed on day +84 after HSCT using a left lobe graft from her 46-year-old mother. Preoperatively, the patient weighted 49.3 kg. The CT volumetry showed the total liver volume of donor was 886 cm^3^, the graft weight was 294 g, and graft volume/standard liver volume ratio was 28%, and the graft-to-recipient weight ratio was 0.59%.

**Figure 1:**
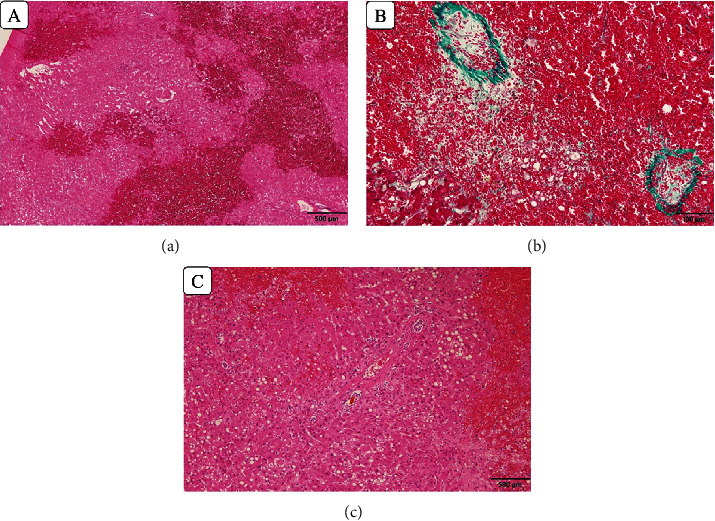
The histology of the explanted liver. (a) Hemorrhage and necrosis in the centrilobular lesion (zone 3). (b) Central vein obliteration. (c) Portal areas are relatively intact.

An enlarged liver with marked congestion and extensive collateral retroperitoneal circulation was found during surgery. Additionally, serosal sclerosis in the small intestine and mesentery indicated that the myeloablative preparative chemoradiotherapy influence extended to the liver. Histopathologic findings showed necrosis and hemorrhage in zone 3 and central vein obliteration, which were compatible with VOD/SOS (Figure 1).

No postoperative surgical complications occurred. A daily granulocyte-stimulating factor was administered to keep the white blood cell level at >5000/*μ*L from postoperative days (POD) 1 to 18. Immunosuppression consisted of steroids and tacrolimus. Mycophenolate mofetil was not used because of myelosuppression. The steroid was started on POD 1, and tacrolimus was administered on POD 5. Immediately after LDLT, restrictive ventilatory impairment with widespread alveolar injury on chest radiography occurred. Cultures or other assays revealed the absence of bacterial, fungal, or viral infection.

**Figure 2:**
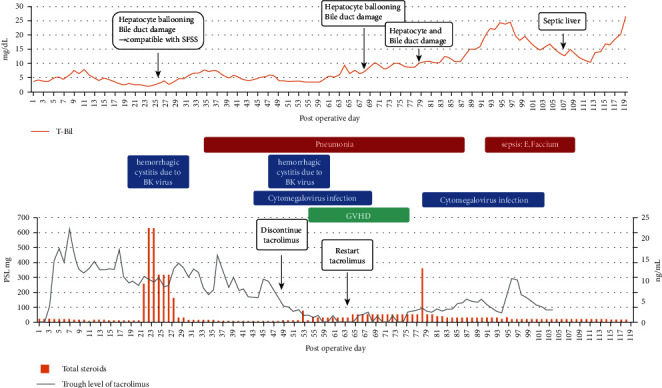
Postoperative course in this patient. The upper graph shows total bilirubin, aspartate aminotransferase, and alanine aminotransferase level after LDLT, as well as liver biopsy findings. The lower band shows the duration of granulocyte-stimulating factor administration and infectious events.

Graft function was favorable until 3 weeks after LDLT, and total bilirubin decreased to 2.0 mg/dL. However, liver enzymes were gradually elevated after the granulocyte-stimulating factor was discontinued. A liver biopsy on POD 26 revealed hepatocyte ballooning and suggested small-for-size syndrome (SFSS) (Figure 2). Subsequently, her respiratory condition deteriorated due to atelectasis from pleural effusion and pneumonia. Several viral infections developed thereafter, including hemorrhagic cystitis due to BK virus and cytomegalovirus infection. Therefore, tacrolimus therapy was discontinued and only prednisolone was administered.

Afterward, skin rashes appeared on her trunk, and a skin biopsy revealed GVHD. Tacrolimus administration was resumed and GVHD improved; however, individualizing the immunosuppressant drug therapy and controlling the infections became very difficult. Infections led to graft dysfunction, and increased ascites caused infections. Several liver biopsies were performed, which showed no signs of rejection, but hepatocyte ballooning and bile duct damage were noticeable. Sepsis developed and she finally died of multiorgan failure (MOF) on day 119 after LDLT.

## 3. Discussion

VOD/SOS is a life-threatening complication following HSCT and is characterized by tender hepatomegaly, elevated serum bilirubin, and weight gain. Its reported mean incidence is 14% of patients following HSCT, but this varies among studies depending on the preparative regimen and diagnostic criteria [4]. The primary insult of this disease is the sinusoidal endothelial cell damage in zone 3 of the hepatic acinus [5]. This causes a circular disorder and nonthrombotic obstruction, which provokes fibrosis and sinusoidal portal hypertension, and finally leads to liver failure [4, 6]. According to the EBMT criteria for severity grading, our patient was classified as having very severe VOD/SOS [7]. Almost all cases of mild or moderate VOD/SOS are self-limiting and resolve within a few weeks, but the mortality rate for severe VOD/SOS is >80% [4].

Historically, VOD/SOS treatment had included supportive care, but defibrotide has been currently highly recommended in Western guidelines. However, its outcome for severe VOD/SOS has not been promising [8, 9].

Defibrotide was not approved in Japan during this case (2019/6-approved). LDLT was decided because the prognosis seemed to be very poor with only conservative treatment, and this patient seemed to be a good candidate for liver transplantation other than graft size. Some reports have indicated performing liver transplantation in patients with VOD/SOS without original disease relapse, as well as evidence of bone marrow engraftment, absence of acute GVHD, and absence of severe other organ failures in those with a good original disease prognosis after HSCT [10]. Our patient fulfilled all of these indications.

There have been 17 reports on liver transplantation for VOD/SOS originating from HSCT (Table 1) [10–23]. Six (35%) patients survived longer than the follow-up period (9 months–8 years). The results have been unsatisfactory, especially in adults (3/15, 20%). In the meantime, three patients (all infants) underwent LDLT, and their day +100 survival rate was 100%. The result that the outcome of pediatric cases was superior to adult cases suggests that LDLT in infants would be preferable to that in adults. However, these results are speculated to be attributable to graft size and the regenerative potential in both liver and bone marrow, which made these results unclear.

Bone marrow progenitor of liver sinusoidal endothelial cells (BM sprocs) was recruited after liver injury and plays an important role in liver regeneration. BM sprocs provide hepatocyte growth factor, which stimulates driving hepatocytes toward proliferation [24]. Therefore, liver regeneration is impaired under BM suppression, which might be consistent with some pediatric patients with acute liver failure simultaneously developing BM failure [25]. Additionally, Lee et al. suggested that BM sprocs originating from the recipient are most important for graft volume restoration in liver transplantation [26]. Therefore, liver regeneration after LDLT could be achieved only when bone marrow function is satisfactory. In adult LDLT cases, graft regeneration is necessary, especially in small graft cases, such as graft volume of <40%. Consistently, the LDLT outcome for VOD/SOS in an infant is preferable to that of an adult as mentioned because ensuring sufficient graft volume in an infant case is easy.

In our case, preoperative bone marrow aspiration achieved engraftment, but lab tests showed pancytopenia despite the daily GCSF administration (Hb of 7.7 g/dL, leukocytes of 3,700/L, and platelets of 49,000/L). This data indicated that our patient was still in the bone marrow recovery phase, and the bone marrow function was inadequate to regenerate a partial liver graft.

Among these 17 reported patients, 10 died of infection and/or MOF, suggesting that infection control is important in such patients. Especially, our patient may be managed with less immunosuppression in liver transplantation after HSCT because, first, our patient received allogeneic HSCT from an HLA-matched sibling donor. HLA allele mismatching is widely known as a significant risk factor for GVHD [27]; thus, our patient had the lowest risk of GVHD. Second, the risk of infections is more in recipients who undergo HSCT compared with those who do not. Immune system recovery following HSCT is a highly dynamic process, which begins with innate immunity resurgence within the first few weeks of HSCT, followed by adaptive immune system resurgence. The complete recovery of the latter may take 2 years or longer [28]. Particularly, our patient suffered from some viral infections; thus, we might be able to reduce steroids.

Once infections emerge, individualizing immunosuppressive drug therapy and controlling infections after the GVHD development is difficult. GVHD and its associated therapies result in profound immunosuppression [29], which could cause infection and exacerbate existing infections. Thus, continuous administration of immunosuppressive drugs even at a minimal dose in cases of active infection is considered important to prevent GVHD.

In summary, this patient was diagnosed with very severe VOD/SOS after HSCT. No other treatment option was available than liver transplantation because conservative therapy was unlikely to improve the condition. LDLT was performed, but finally, this patient died of MOF due to infections. A low dose of immunosuppression should be managed in liver transplantation after HSCT because these patients may be vulnerable to infection. After all, immune system recovery takes a long time. We had no choice but to perform LDLT with a small graft; however, securing a sufficient graft volume is important in liver transplantation for bone marrow failure because normal liver regeneration cannot be expected.

In conclusion, severe VOD/SOS is associated with a very poor prognosis. The outcome of liver transplantation for VOD/SOS has not been satisfactory but should be considered as a therapeutic option in case a sufficient graft volume can be secured because it may provide long-term survival if successful.

## Figures and Tables

**Table 1:** Data of previous reports on liver transplantation for VOD/SOS originating from HSCT.

	LT type	Age/sex	Conditioning regimens	Time to LT (day)	Follow-up period status		Cause of death
Case 1 (11)	Deceased	55/F	CY/BU	42	54 days	Death	Infection
Case 2 (12)	Deceased	49/M	CY/BU	31	31 days	Death	MOF
Case 3 (13)	Deceased	47/M	CY/BU	41	60 days	Death	Infection/MOF
Case 4 (14)	Deceased	43/M	Etoposide/CY/carmustine	22/44(1^st^/2^nd^)	70 days	Death	Pneumonia, ARDS/TTP
Case 5 (13)	Deceased	39/F	CY/BU	79	62 days	Death	Aspergillosis/intracerebral hemorrhage/MOF
Case 6 (15)	Deceased	38/M	CY/BU	23	8 years	Alive	
Case 7 (16)	Deceased	35/M	CY/TBI	43	9 months	Alive	
Case 8 (17)	Deceased	34/F	CY/BU	21	1 year	Alive	
Case 9 (18)	Deceased	34/F	CY/BU	35	42 days	Death	Interstitial pneumonitis/MOF
Case 10 (19)	Deceased	33/M	CY/BU	36	3 days	Death	Cerebral edema
Case 11 (19)	Deceased	32/F	CY/TBI	39	30 days	Death	Pneumonia
Case 12 (19)	Deceased	31/F	CY/BU	25	213 days	Death	Liver failure
Case 13 (20)	Deceased	25/M	CY/BU	32	6 months	Death	Pneumocystis pneumonia
Case 14 (21)	Deceased	23/M	CY/BU	80	140 days	Death	Infection
Case 15 (22)	Living	2 m/F	CY/TBI	33	9 months	Alive	
Case 16 (23)	Living	1/F	CY/BU	84	29 months	Alive	
Case 17 (10)	Living	11 m/M	CY/BU/TBI	42	17 months	Alive	
Our case	Living	16/F	CY/etoposide/TBI	84	119 days	Death	Sepsis/MOF

LT: Liver transplantation; Cy: Cyclophosphamide; BU: Busulfan; TBI: Total body irradiation; MOF: Multiorgan failure; ARDS: Acute respiratory distress syndrome; TTP: Thrombotic thrombocytopenic purpura.

## Abbreviations

B-ALL: B-cell precursor acute lymphoblastic leukemia
BU: Busulfan
CY: Cyclophosphamide
EBMT: European Society for Blood and Marrow Transplantation
GVHD: Graft-versus-host disease
HSCT: Hematopoietic stem cell transplantation
LDLT: Living donor liver transplantation
MOF: Multiorgan failure
POD: Postoperative day
rhTM: Recombinant human soluble thrombomodulin
SFSS: Small-for-size syndrome
SOS: Sinusoidal obstruction syndrome
TBI: Total body irradiation
VOD: Venoocclusive disease.
